# Exergoeconomic Analysis of Power Plants: Impact of Exergy Destruction Due to Entropy Generation on Unit Cost of Electricity

**DOI:** 10.3390/e28101097

**Published:** 2026-10-08

**Authors:** Yungpil Yoo, Seok-Ho Seo, Si-Doek Oh, Ho-Young Kwak

**Affiliations:** 1 Department of Climate Change Energy Engineering, Yonsei University, Seoul 03722, Republic of Korea; yyp@besico.co.kr (Y.Y.); ohsidoek@besico.co.kr (S.-D.O.); 2 Blue Economy Strategy Institute Co., Ltd., Seocho-gu, Seoul 06640, Republic of Korea; shseo@besico.co.kr; 3 Department of Mechanical Engineering, Chung-Ang University, Seoul 06974, Republic of Korea

**Keywords:** unit cost of electricity, power plant, exergoeconomic methods, exergy destruction, lost cost

## Abstract

Various exergy-based exergoeconomic methods have been developed to evaluate the production cost of electricity in power systems. One of the unresolved issues in the field of exergoeconomics is that different exergoeconomic analysis methods can yield different unit costs of power production. In this study, the unit cost of electricity generation is comprehensively analyzed using three representative approaches: the Moran method, the specific exergy costing (SPECO) method, and the Modified Productive Structure Analysis (MOPSA) method. The objective of this study is to identify a condition under which different exergoeconomic methods yield the same production cost for a power plant. The results show that power production costs are obtained using all three exergoeconomic methods when the costs associated with exergy destruction are appropriately incorporated in the cost-balance equation. In other words, under this condition, the cost-balance equations of SPECO and MOPSA for the entire power plant reduce to the Moran equation. These findings indicate that the critical issue in exergoeconomic analysis for determining the unit cost of products is whether the cost of exergy destruction is explicitly or implicitly accounted for. Furthermore, the numerical results demonstrate that all input cost rates, including equipment investment, operation and maintenance, and fuel costs, are fully recovered through the cost flow rates of the products. It is also shown that the cost flow associated with exergy destruction ultimately appears as the cost flow of waste streams.

## 1. Introduction

Exergy analysis of thermal systems is a powerful tool for evaluating the quality and utility of energy beyond the concept of energy based on the first law of thermodynamics. The concept of flow exergy in power plants and thermal systems was addressed by Howell [1] and Bejan [2] in the late 1980s. Thanks to this pioneering research, it has been revealed that exergy analysis can quantify the entropy generated within power plant and thermal system components and calculate the lost work in the components. In addition, through exergy analysis, it has become possible to design power plants and chemical process plants and to analyze and simulate system performance [3].

Since Tribus [4] initiated the development of exergoeconomics in the 1960s, which was further advanced by Frangopoulos [5] in the late 1980s, von Spakovsky and Evans [6], Valero et al. [7,8], Lazzaretto and Tsatsaronis [9], and Kwak et al. [10] in the 1990s conducted systematic research on exergoeconomics and developed various exergoeconomic methods. However, although various methods are used in exergoeconomics based on exergy analysis, each method yields different unit costs for products. Among the exergoeconomic analysis methods, the specific exergy costing (SPECO) method by Lazzaretto and Tsatsaronis [9,11] and the Modified Productive Structure Analysis (MOPSA) method by Kwak et al. [10] have been applied to the analysis, optimal design, and unit cost calculation of various power plants. SPECO has been applied to determine changes in design parameters of the copper–chlorine thermochemical cycle for hydrogen production [12], to obtain the unit cost of electricity produced from a diesel engine [13], and to obtain the unit cost of farine, clinker, and cement produced from a cement plant [14]. Also, the SPECO method was used in the study of exergoeconomics for a biomass-to-energy integrated thermal power plant [15]. Meanwhile, the MOPSA method by Kim et al. was applied to obtain the electricity unit costs of gas turbines and steam turbines of a 500 MW combined cycle plant [10].

Each exergoeconomic method relies on different grounds and assumptions in establishing the cost-balance equation, and therefore calculates the unit cost of products produced at the power plant differently. Several researchers have compared the SPECO and MOPSA methods in exergy cost estimation [16,17]. Uysal et al. [16] calculated the unit cost of electricity produced at a 160 MW coal-fired power plant using the SPECO and MOPSA methods through detailed analysis. The total equipment cost, operating hours, and lifespan of the power plant were $91.67 million, 8000 h, and 25 years, respectively (2017). The chemical exergy of the coal used is approximately 362.3 MW, the power produced is 143.3 MW, and the exergy destruction rate of the entire system is approximately 217.8 MW. SPECO estimated the unit cost of power produced by the system at $12.14 per GJ, while MOPSA estimated it at $14.06 per GJ. The unit cost of work lost due to exergy destruction calculated by MOPSA is approximately −$1.64/GJ. Yilmaz [17] performed a thermoeconomic cost analysis for a binary geothermal power plant using the methods of Moran, SPECO, and MOPSA. This power plant produces electricity by introducing heat obtained from geothermal water entering at a temperature of 160.1 °C and exiting at 62 °C at a rate of 168.9 kg per second into an organic Rankine cycle. The exergy input and power output of the geothermal plant were calculated to be 20.174 MW and 9.543 MW, respectively. The geothermal power plant spent a total of $8.13 million on equipment costs, and the annual operation and maintenance costs are $510,000. The unit cost of electricity obtained by SPECO, MOPSA, and Moran from the system was $10.22/GJ, $12.13/GJ, and $9.88/GJ, respectively. This study did not present the unit cost of lost work due to exergy destruction.

As confirmed by the comparative analysis results of SPECO and MOPSA, for the CGAM cogeneration power plant, coal-fired power plant, and geothermal power plant, the unit cost of electricity calculated by SPECO is 8–16% lower than the cost calculated by MOPSA. In a study on the role of waste costs in coal-fired power plants and CGAM cogeneration power plants, Uysal and Kwak [18] found that in order to obtain the appropriate unit cost of the products produced at the power plant, the waste costs removed from the dissipation unit must be allocated to the power plant components. In the SPECO method, the cost of waste going to the dissipation equipment such as condenser is not allocated to the plant’s components, so the unit cost of electricity produced by CGAM cogeneration power plant, coal-fired power plant, and geothermal power plants is lower than the value calculated by the MOPSA method. Hou et al. [19] calculated the unit cost of lost work assuming it was equal to the fuel unit cost in an optimization study of combined heat and power systems including gas turbine cycles, supercritical CO_2_ compression cycles, steam turbine cycles, and organic Rankine cycles. However, the results showed that the cost of lost work in air compressors was greater than the cost flow of the total facility investment, and the cost of lost work due to the total equipment was more than twice the cost of the facility investment. As such, the MOPSA method can accurately estimate production costs by accounting for the cost of lost work associated with exergy destruction.

In this study, the unit costs of products in gas turbine power plants, cogeneration plants, and combined cycle power plants were evaluated using the SPECO method after appropriately allocating the cost associated with exergy destruction to individual system components. The resulting product unit costs were found to be in close agreement with those obtained using the MOPSA method. Meanwhile, the MOPSA method, which implicitly accounts for the cost of exergy destruction, yielded product unit costs identical to those calculated by the Moran method. Since the Moran method is a product cost evaluation approach based on the second law of thermodynamics, it can serve as a benchmark for assessing the validity of product unit costs calculated for power generation systems. Furthermore, the environmental impact associated with the destruction of exergy discharged as residues across the system boundary was also examined.

## 2. Combined Cycle Plant

**Figure 1:**
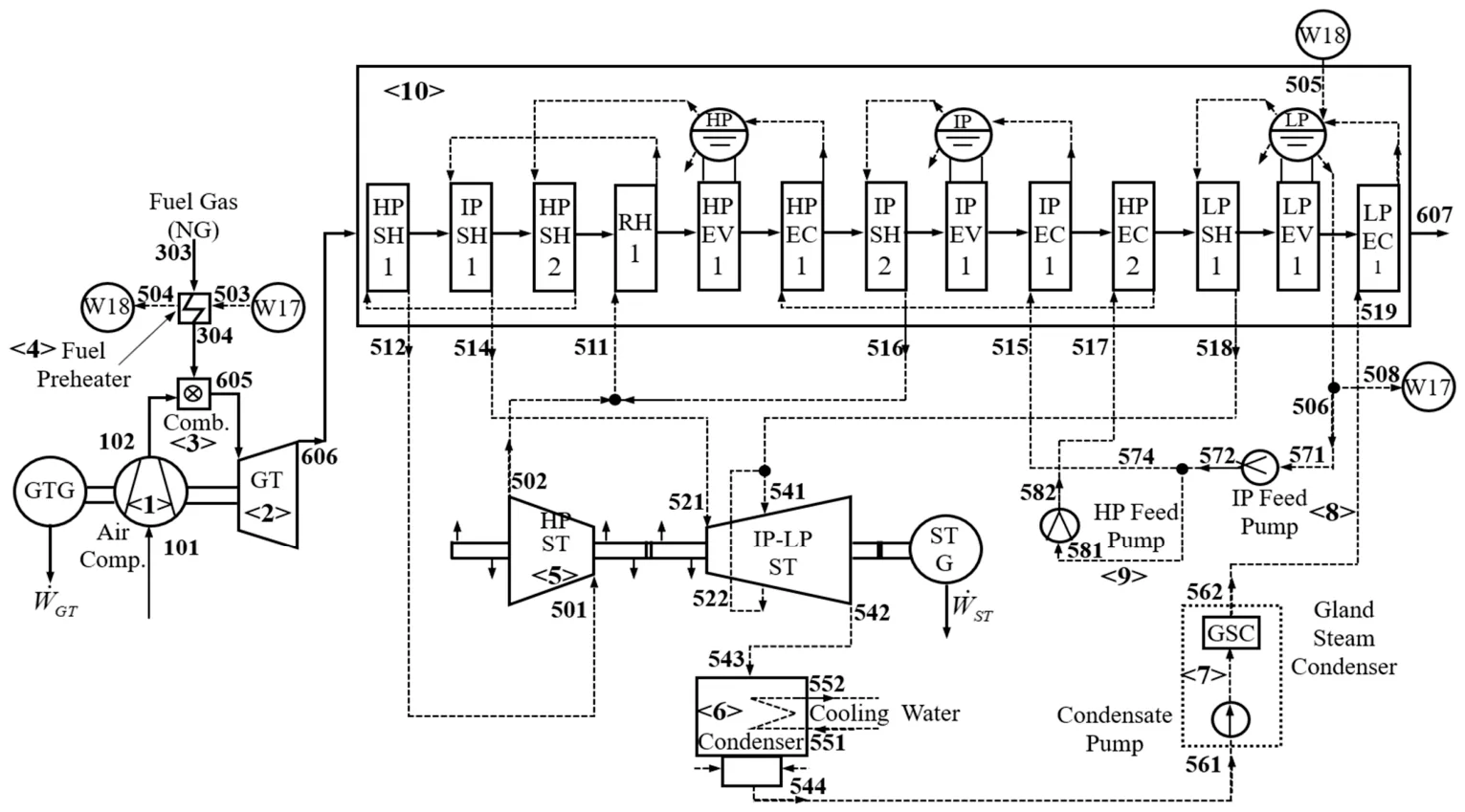
Schematic of a combined cycle plant.

In Figure 1, a schematic of the 300 MW combined cycle power plant system used as the reference plant shows every state point accounted for in this analysis. The schematic shows ten components: <1> air compressor (AC), <2> gas turbine (GT), <3> combustor (COM), <4> fuel preheater (FPH), <5> steam turbines (ST), <6> condenser (CON), <7> condensate pump (CP), <8,9> two feed pumps (FP-A, FP-B), <10> heat recovery steam generator (HRSG). As can be seen in Figure 1, the high-pressure superheater (HP-SH [1]) supplies steam to the high-pressure steam turbine (HP-ST) and the intermediate-pressure superheater (IP-SH 1) supplies steam to the intermediate-pressure steam turbine (IP-ST). Meanwhile, the steam from the IP-ST and the low-pressure superheater (LP-SH 1) flows to the low-pressure steam turbine (LP-ST). The steam exiting the LP-ST is condensed in the condenser and then flows into the low-pressure economizer (LP-EC 1) through the condensate pump. Water from the low-pressure boiler (LP) flows into the high-pressure economizer (HP-EC 2) and intermediate-pressure economizer (IP-EC 1) through the feed pumps, <8> and <9>, respectively. Steam from the high-pressure steam turbine (HP-ST) combines with steam from the intermediate-pressure superheater (IP-SH 2) and enters the reheater (RH 1). The mole flow rates of fuel, air, and steam (or water) and thermodynamic state quantities such as temperature and pressure for a 300 MW power plant based on design/initial operation data from a power company [20] are shown in Table 1. The data presented in Table 1 corresponds to the 100% load condition, where efficiency is highest. Using the data, thermodynamic properties including enthalpy and entropy at all state points were calculated using polynomial correlations [21] fitted to the thermophysical data in the JANAF table [22]. The thermophysical properties of water and steam were calculated using data at the state point with the equations recommended by the International Formulation Committee (IFC) [23]. Calculated values of enthalpy, entropy, and exergy flow rates are also given in this table. The state point is indicated by three numbers, where the first number is 1 for air flow, 3 for fuel flow, 5 for water or steam flow, and 6 for flue gas flow. This combined cycle power plant can be utilized as a gas turbine power plant or a cogeneration plant that produces electricity or electricity and steam by selecting appropriate system boundaries.

## 3. Energy Conservation and Exergy-Balance Equations

This section presents energy conservation and exergy-balance for each component of the combined cycle plant. The energy conservation for each component can be written as [21]

(1) $${\dot{Q}}_{\mathrm{cv}}+\sum_{\mathrm{in}}{\dot{H}}_{i}=\sum_{\mathrm{out}}{\dot{H}}_{i}+{\dot{W}}_{\mathrm{cv}}$$

In Equation (1), ${\dot{H}}_{i}={\dot{m}}_{i}h_{i}$, h is the enthalpy per unit mass, ${\dot{Q}}_{\mathrm{cv}}$ is the heat interaction with the environment, and ${\dot{W}}_{\mathrm{cv}}$ is the work rate in the component. Of course, the law of conservation of mass must hold for each component.

Based on the first and second laws of thermodynamics, the following exergy-balance equation of each component can be derived [3]:

(2) $${\dot{E}}_{x}^{\mathrm{CHE}}+\left(\sum_{\mathrm{inlet}}{\dot{E}}_{x}^{T}-\sum_{\mathrm{outlet}}{\dot{E}}_{x}^{T}\right)+\left(\sum_{\mathrm{inlet}}{\dot{E}}_{x}^{P}-\sum_{\mathrm{outlet}}{\dot{E}}_{x}^{P}\right)+T_{o}\left(\sum_{\mathrm{inlet}}{\dot{S}}_{i}-\sum_{\mathrm{outlet}}{\dot{S}}_{i}+\frac{{\dot{Q}}_{\mathrm{cv}}}{T_{o}}\right)={\dot{E}}_{x}^{W}$$

In Equation (2), the superscripts *CHE*, *T*, *P*, and *W* represent chemical, thermal, and mechanical exergy and work, respectively. The fourth term on the left side represents the negative value of the exergy destruction rate, defined by the Gouy–Stodola theorem as follows [2].

(3) $${\dot{E}}_{x}^{D}=T_{o}{\dot{S}}_{\mathrm{gen}}=-T_{o}\left(\sum_{\mathrm{inlet}}{\dot{S}}_{i}-\sum_{\mathrm{outlet}}{\dot{S}}_{i}+\frac{{\dot{Q}}_{\mathrm{cv}}}{T_{o}}\right)$$

The exergy destruction rate can be determined from the heat transfer rate obtained through the energy conservation given in Equation (1) and the difference between entropy inflow and outflow rates listed in Table 1. It should be noted that the exergy destruction rate is not an arbitrary quantity; rather, it is uniquely determined by the thermodynamic state and operating conditions of the system. Accordingly, the exergy-balance equation can also be expressed without decomposing the exergy stream into thermal and mechanical exergy components, as follows [16]:

(4) $${\dot{E}}_{x}^{\mathrm{CHE}}+\left(\sum_{\mathrm{inlet}}{\dot{E}}_{x,i}-\sum_{\mathrm{outlet}}{\dot{E}}_{x,j}\right)={\dot{E}}_{x}^{W}+{\dot{E}}_{x}^{D}$$

Exergy flow rate is expressed as exergy per unit mass as follows.

(5a) $${\dot{E}}_{x}=\dot{m}e_{x}$$

Exergy for material flow can be defined as follows based on reference temperature (T_ref_) and reference pressure (P_ref_):

*e_x_* = *h*(*T*,*P*) − *h_ref_*(*T_ref_*,*P_ref_*) − *T_o_*[*s*(*T*,*P*) − *s_ref_*(*T_ref_*,*P_ref_*)] (5b)

The reference temperature and pressure are taken as 298.15 K and 1 atm, respectively, in this study. The exergy per unit mass (*e_x_*) can be decomposed into thermal exergy, which is associated with temperature, and mechanical exergy, which is associated with pressure [24]. Detailed procedures for evaluating the thermal and mechanical exergy components are provided by Uysal et al. [16]. The exergy-balance equations for each component are shown in Table 2. In addition, the detailed entropy destruction rates in the exergy-balance equation obtained from the Gouy–Stodola theorem for each component, which are required for the exergoeconomic analysis of the combined cycle power plant, are also shown in the third column of Table 2.

## 4. Thermoeconomic Analysis

### 4.1. Moran’s Method

Moran’s method does not consider detailed exergy balances for power plant components, but only the exergy-balance of the entire system. Assuming that the power plant produces only one product, electricity, the power plant’s exergy-balance can be expressed as follows.

(6) $${\dot{E}}_{x}^{\mathrm{CHE}}={\dot{E}}_{x}^{W}+\sum_{k}{\dot{E}}_{x,k}^{D}$$

The corresponding overall exergy cost-balance equation may be written as

(7) $$c_{o}{\dot{E}}_{x}^{\mathrm{CHE}}+\sum_{k}{\dot{Z}}_{k}=c_{W}{\dot{E}}_{x}^{W}$$

In Equation (7), *c_O_* and *c_W_* are the unit costs of fuel and electricity, respectively, and ${\dot{Z}}_{k}$ is the cost flow per unit time. Several exergy flows, including entropy flows inside the plant, are not included in Equation (7) because they are internal parameters [25]. Equation (7) represents the product cost resulting from the second law efficiency of the plant [10].

### 4.2. Specific Exergy Costing Method (SPECO)

The SPECO method obtains the corresponding exergy cost-balance equation using the exergy-balance equation given in Equation (4). The SPECO method imposes different exergoeconomic unit costs for different material flows under different state conditions.

(8) $$c_{o}{\dot{E}}_{x}^{\mathrm{CHE}}+\left(\sum_{\mathrm{inlet}}c_{i}{\dot{E}}_{x,i}-\sum_{\mathrm{outlet}}c_{j}{\dot{E}}_{x,j}\right)+{\dot{Z}}_{<k>}=c_{w}{\dot{E}}_{x,<k>}^{W}+c_{S}{\dot{E}}_{x,<k>}^{D}$$

In the original SPECO method [9,11], the cost of exergy destruction was not considered; however, in this study, a unit cost of exergy destruction is assigned in the cost-balance Equation (8) for each component as shown in Equation (8).

In the SPECO method, the following “fuel rule” and “product rule” are used in the cost-balance equation for each component of a combined cycle power plant to find the exergoeconomic unit cost of the material flow [9,16].

*Fuel rule*: When the substance (fuel) entering a device is equal to the product exiting the device, the unit cost of that substance is the same.

*Product rule*: The unit cost of products produced by the same equipment is the same. For example, the unit cost of water or steam from the HRSG is the same.

The cost-balance equations for combined cycle power plant components using the SPECO method are shown in Table 3. In addition, the fuel and product rules applicable to the cost-balance equations for each component are listed in the third column of the table.

By applying these fuel and product rules to each component listed in Table 3, the following 10 equations containing the 10 unknown unit costs can be obtained for each component in the combined cycle power plant.

(9) $$c_{1}{\dot{E}}_{x,102}+c_{3}E_{x,<\mathrm{AC}>}^{W}=-c_{S}E_{x,<\mathrm{AC}>}^{D}+{\dot{Z}}_{<\mathrm{AC}>}$$

(10) $$-c_{1}{\dot{E}}_{x,102}-c_{4}{\dot{E}}_{x,304}+c_{2}{\dot{E}}_{x,605}=-c_{S}{\dot{E}}_{x,<\mathrm{COM}>}^{D}+{\dot{Z}}_{<\mathrm{COM}>}$$

(11) $$c_{2}\left({\dot{E}}_{x,606}-{\dot{E}}_{x,605}\right)+c_{3}{\dot{E}}_{x,<\mathrm{GT}>}^{W}=-c_{S}{\dot{E}}_{x,<\mathrm{GT}>}^{D}+{\dot{Z}}_{<\mathrm{GT}>}$$

(12) $$c_{4}{\dot{E}}_{x,304}+c_{10}\left({\dot{E}}_{x,504}-{\dot{E}}_{x,503}\right)=-c_{S}{\dot{E}}_{x,<\mathrm{FPH}>}^{D}+{\dot{Z}}_{<\mathrm{FPH}>}+c_{o}{\dot{E}}_{x}^{\mathrm{CHE}}$$

(13) $$c_{10}\left[\left({\dot{E}}_{x,502}-{\dot{E}}_{x,501}\right)+\left({\dot{E}}_{x,522}-{\dot{E}}_{x,521}\right)+\left({\dot{E}}_{x,542}-{\dot{E}}_{x,541}\right)\right]+c_{5}{\dot{E}}_{x,<\mathrm{ST}>}^{W}=-c_{S}{\dot{E}}_{x,<\mathrm{ST}>}^{D}+{\dot{Z}}_{<\mathrm{ST}>}$$

(14) $$c_{10}\left({\dot{E}}_{x,544}-{\dot{E}}_{x,543}\right)+c_{6}{\dot{E}}_{x,552}=-c_{S}{\dot{E}}_{x,<\mathrm{CON}>}^{D}+{\dot{Z}}_{<\mathrm{CON}>}$$

(15) $$-c_{10}{\dot{E}}_{x,561}+c_{7}{\dot{E}}_{x,562}+c_{5}{\dot{E}}_{x,<\mathrm{CP}>}^{W}=-c_{S}{\dot{E}}_{x,<\mathrm{CP}>}^{D}+{\dot{Z}}_{<\mathrm{CP}>}$$

(16) $$-c_{10}{\dot{E}}_{x,571}+c_{8}{\dot{E}}_{x,572}+c_{5}{\dot{E}}_{x,<\mathrm{FPA}>}^{W}=-c_{S}{\dot{E}}_{x,<\mathrm{FPA}>}^{D}+{\dot{Z}}_{<\mathrm{FPA}>}$$

(17) $$-c_{10}{\dot{E}}_{x,581}+c_{9}{\dot{E}}_{x,582}+c_{5}{\dot{E}}_{x,<\mathrm{FPB}>}^{W}=-c_{S}{\dot{E}}_{x,<\mathrm{FPB}>}^{D}+{\dot{Z}}_{<\mathrm{FPB}>}$$

(18) $$\begin{array}{c} c_{2}\left({\dot{E}}_{x,607}-{\dot{E}}_{x,606}\right)-c_{7}{\dot{E}}_{x,515}-c_{8}{\dot{E}}_{x,517}\\ -c_{9}{\dot{E}}_{x,519}-c_{10}\left({\dot{E}}_{x,505}+{\dot{E}}_{x,511}\right)\\ +c_{10}\left({\dot{E}}_{x,506}+{\dot{E}}_{x,508}+{\dot{E}}_{x,512}+{\dot{E}}_{x,514}+{\dot{E}}_{x,516}+{\dot{E}}_{x,518}\right)=-c_{S}{\dot{E}}_{x,<\mathrm{HRSG}>}^{D}+{\dot{Z}}_{<\mathrm{HRSG}>} \end{array}$$

Solving the above 10 cost-balance equations yields the 10 unknown unit costs. The unit costs newly defined in Equation (9) to Equation (18) are shown in Table 6a in Section 5.

Summing all cost-balance equations from Equation (9) to Equation (18) yields the following equation.

(19) $$c_{3}\left({\dot{E}}_{x,<\mathrm{AC}>}^{W}+{\dot{E}}_{x,<\mathrm{GT}>}^{W}\right)+c_{5}\left({\dot{E}}_{x,<\mathrm{ST}>}^{W}+{\dot{E}}_{x,<\mathrm{CP}>}^{W}+{\dot{E}}_{x,<\mathrm{FPA}>}^{W}+{\dot{E}}_{x,<\mathrm{FPB}>}^{W}\right)+\;\left[c_{2}{\dot{E}}_{x,607}+c_{6}{\dot{E}}_{x,552}+c_{S}\sum_{k}{\dot{E}}_{x,<k>}^{D}\right]=\sum_{k}{\dot{Z}}_{<k>}+c_{o}{\dot{E}}_{x}^{\mathrm{CHE}}$$

Equation (19) reduces to the Moran equation only when the third term in parentheses disappears. This indicates that the waste cost flow rate flowing from the dissipation unit into the environment should be considered as the loss cost flow rate due to exergy destruction. One may estimate the unit cost of lost work using the following relation for the combined cycle plant.

(20) $$c_{2}{\dot{E}}_{x,607}+c_{6}{\dot{E}}_{x,552}+c_{S}\sum_{k}{\dot{E}}_{x<k>}^{D}=0$$

### 4.3. Modified Productive Structure Analysis (MOPSA)

The Modified Productive Structure Analysis (MOPSA) method [10] is an exergoeconomic approach in which different unit costs are assigned to the various exergy forms appearing in the exergy-balance equation (Equation (2)). Let C_O_, C_T_, C_P_, C_S_, and C_W_ represent the unit costs of fuel exergy, thermal exergy, mechanical exergy, lost work, and work (electricity), respectively. Applying these unit costs to Equation (2) results in the following exergy cost-balance equation:

(21) $${\dot{E}}_{x}^{\mathrm{CHE}}c_{0}+\left(\sum_{\mathrm{inlet}}{\dot{E}}_{x,i}^{T}-\sum_{\mathrm{outlet}}{\dot{E}}_{x,i}^{T}\right)c_{T}+\left(\sum_{\mathrm{inlet}}{\dot{E}}_{x,i}^{P}-\sum_{\mathrm{outlet}}{\dot{E}}_{x,i}^{P}\right)c_{P}+T_{0}\left(\sum_{\mathrm{inlet}}{\dot{S}}_{i}-\frac{\sum_{\mathrm{outlet}}{\dot{S}}_{j}+{\dot{Q}}_{\mathrm{cv}}}{T_{o}}\right)c_{s}+{\dot{Z}}_{k}={\dot{E}}_{x}^{W}c_{W},$$

The exergy cost-balance equation described above is generally formulated by analyzing the differences between the exergy flows entering and leaving each component. This approach is particularly useful for determining the cost associated with exergy transformations that occur as a material stream passes through a device. In the cost-balance equation of each component, a new unit cost, denoted by a Gothic symbol, is assigned to the product representing the specific function or characteristic of the component, as expressed below. The cost-balance equations for all components of the combined cycle power plant using MOPSA are shown in Table 4. As can be seen from the 10 cost-balance equations for the 10 components, 17 unknown unit costs appear, so 7 additional equations are needed to solve the cost-balance equations.

Six additional equations can be derived from the junctions of different exergy flows.

These include the thermal and mechanical exergy flows of air, fuel, and flue gas; the thermal and mechanical exergy flows of water and steam; the junction associated with the work produced by the gas turbine; and the junction associated with the work produced by the steam turbine. Finally, an additional equation is obtained from the boundary component of the power plant. The boundary component has the unique role of discharging waste streams from the plant to the environment in the MOPSA method. Therefore, all waste exergy leaving the system through the boundary is considered to have an environmental impact.

By solving the 10 cost-balance equations for the components and 7 auxiliary equations simultaneously, 17 unknown unit costs can be obtained. The sum of all cost-balance equations listed in Table 4 reduces to the Moran equation given in Equation (7) because the following equation is satisfied.

(22) $$\sum_{k}{\dot{E}}_{x,<k>}^{D}=T_{o}\left({\dot{S}}_{607}-{\dot{S}}_{101}-{\dot{S}}_{103}+{\dot{S}}_{552}-{\dot{S}}_{551}-\sum_{k}{\dot{Q}}_{<k>}/T_{o}\right)$$

Equation (22) indicates that all exergy destruction within the power plant equipment is disposed of as waste to the environment through the boundary.

### 4.4. Evaluation of Cost Flow Rate

In the cost-balance equations appearing in Equations (7), (8) and (19), ${\dot{Z}}_{k}$ represents the cost flow per unit time, which includes both the initial purchase cost and the operating cost of the k-th component. It is calculated using the following equation [16,26]:

(23) $${\dot{Z}}_{k}=\frac{\varphi_{k}\cdot {\dot{C}}_{\mathrm{ini}}}{\delta_{k}}$$

In Equation (23), *φ_k_* and *δ_k_* are the maintenance cost factor and annual operation time, respectively. In Equation (24), ${\dot{C}}_{\mathrm{ini}}$ is the initial investment of the component converted into an annual equivalent cost, which is given as follows.

(24) $${\dot{C}}_{\mathrm{ini}}=\left[C_{\mathrm{ini}}-\mathrm{SV}\cdot \mathrm{PWF}(i,n)\right]\cdot \mathrm{CRF}(i,n)$$

In Equation (24), SV is the salvage value after n years of the component’s life and PWF and CRF denote the present value factor and the capital recovery factor, respectively.

## 5. Calculation Results and Discussion

The heat transfer interaction between each component and the environment can be calculated using the enthalpy flow rate at the corresponding state point from Equation (1), and this amount is shown in the sixth column of the exergy balance for each component shown in Table 5. In this table, a minus sign indicates the amount of heat flowing into the component, and a plus sign indicates the amount of heat flowing out of each component. Therefore, the plus sign of $-{\dot{Q}}_{<k>}$ indicates an outflow of heat from the corresponding component and consequently increases the exergy destruction of the component as can be seen from the Gouy–Stodola theorem in Equation (3). Heat is transferred from the surrounding environment to the components only in the condenser.

As clearly seen in the exergy cost-balance equation for the combustor in Table 5, the enthalpy of combustion *LHV*_<*COM*>_ must be included in the energy conservation equation and the exergy-balance equation of the MOPSA method. The natural gas supplied to the combustor consists of 90.0% methane, 6.1% ethane, 2.6% propane, and 1.3% butane. The fifth column of the exergy-balance table (Table 5) represents the exergy destruction rate due to the difference between the inlet and outlet entropy flows, and the sixth column represents exergy destruction due to heat transfer interactions as previously discussed. The magnitude of exergy destruction due to heat transfer interactions (194.9 MW) is comparable to the magnitude of exergy destruction due to changes in entropy flow (210.8 MW). The total exergy destruction rate for the combined cycle plant is approximately 405.7 MW. The amount of electricity produced by the gas turbine and steam turbine is approximately 429.4 MW and 81.6 MW, respectively, and the air compressor consumes 178.9 MW of the electricity produced by the gas turbine. In this table, the exergy rate of the product is represented by a positive value, while the exergy rate of the resource is represented by a negative value.

The following data were used to obtain the capital cost flow rate per unit time for each component shown in Equation (23). The initial investment costs for the air compressor, combustor, gas turbine, fuel preheater, steam turbine, condenser, condensate pump, two feedwater pumps, and heat recovery steam generator are $20.5 million, $2.4 million, $17.5 million, $1.5 million, $23.0 million, $3.9 million, $280,000, $380,000, $540,000, and $8.5 million, respectively. The total initial investment cost for the combined cycle power plant in question is approximately $78.5 million, which represents about 65% of the $120.7 million total initial investment cost for a 500 MW combined cycle power plant built in Korea in 2024. The investment cost of a 300 MW combined cycle power plant is obtained by the following cost to capacity method [27].

(25) $$\frac{C_{2}}{C_{1}}={\left(\frac{Q_{2}}{Q_{1}}\right)}^{X}$$

Here, C_1_ and C_2_ represent the costs of the known equipment 1 and the unknown equipment 2, respectively, while Q_1_ and Q_2_ denote the capacities of the known equipment 1 and 2. Additionally, X is the scaling exponent, which is approximately 0.8 for combined cycle gas turbine power plants [27]. The initial investment cost of a 300 MW power plant, calculated using Equation (25), amounts to approximately 65% of the investment cost of a 500 MW power plant. Construction costs were not included in the data. Applying an annual operating time of 8000 h, an equipment lifespan of 25 years, an annual interest rate of 10%, and a depreciation rate of 12% after 25 years [16], the input cost flow per second for the combined cycle plant, including maintenance, operating costs, and fuel costs, is approximately $13.84/s. The unit fuel cost of natural gas was set at $20.0/GJ in this calculation.

Using the cost flow rate of each component calculated from Equation (23), and the exergy values at the state points of the combined cycle power plant listed in Table 1, Equations (9)–(18) for the SPECO method can be solved to obtain the exergy unit cost at various state points of the combined cycle power plant and the unit cost of electricity produced by the gas turbine and steam turbine. The results by SPECO without considering the cost of loss due to 405.7 MW of exergy destruction are shown in Table 6a. The unit production cost of electricity for gas turbines (*c_W_*) and steam turbine (*c_DW_*) are $25.55/GJ and $35.63/GJ, respectively.

Meanwhile, the unit cost of various exergy flows and the unit cost of power production using gas and steam turbines can be obtained by solving the exergy cost-balance equations obtained by MOPSA, as shown in Table 4. The unit production cost of electricity for gas turbines (*c*_2*W*_) and steam turbines (*c*_3*W*_) by the MOPSA method are $26.96/GJ and $37.68/GJ, respectively, as shown in Table 6b. The power production costs of gas and steam turbines calculated using the MOPSA method are slightly higher than the power generation costs calculated using the SPECO method. This is because the MOPSA method inherently takes into account the cost of losses (*c_S_*) due to exergy destruction, which is approximately −$0.24/GJ, as shown in Table 6b.

**Table 6:** (**a**) Unit costs of material flow at various state points in the combined cycle power plant by SPECO method without considering the lost cost due to exergy destruction (Unit is $/GJ). (**b**) Unit costs of various exergy flows in the cost-balance equations for the combined power plant by MOPSA method (Unit is $/GJ).

**(a)**				
***c*_1_(*c*_102_)**	***c*_2_(*c*_605_)**	***c*_3_(*c_W_*)**	***c*_4_(*c*_304_)**	***c*_5_(*c_DW_*)**
28.05	24.83	25.55	2073.0	35.63
***c*_6_(*c*_552_)**	***c*_7_(*c*_562_)**	***c*_8_(*c*_572_)**	***c*_9_(*c*_582_)**	***c*_10_(*c*_512_)**
28.28	67.91	34.04	34.64	31.88
**(b)**				
***c*_1*P*_**	***c*_2*W*_**	***c*_3*T*_**	***c*_4*T*_**	***c*_5*DW*_**
33.24	26.96	0.30	41588.2	37.68
***c*_6*DT*_**	***c*_7*DP*_**	***c*_8*DP*_**	***c*_9*DP*_**	***c*_10*DT*_**
7.70	111.17	97.45	53.31	34.32
***c_T_***	***c_P_***	***c_DT_***	***c_DP_***	***c_W_***
24.0	33.24	33.61	65.04	26.96
***c_DW_***	***c_S_***			
37.68	−0.24			

The MOPSA method also provides the cost flow rates of exergy entering and exiting each component, the investment cost flow rates of each component, and the loss cost flow rates due to exergy losses, as shown in Table 7a for the combined cycle plant. In this case, the lost cost due to exergy destruction amounts to about 1% of the input cost flow rate due to capital expenditure, operation, maintenance, and fuel cost. The investment cost flow rate of $9.791/s is almost entirely recovered through the power generation cost flow rate of $9.790/s. On the other hand, a loss cost flow rate of $0.104/s can be seen as the cost flow rate of thermal and mechanical exergy released into the environment through the boundary. The same sign convention for the cost flow rates related to products and resources was used as the case of exergy balances shown in Table 5. As can be seen in Table 7a, the loss cost flow rate in each component turns out to be negative, which indicates that the loss cost flow due to exergy destruction increases the operational burden of the plant, similar to the investment cost. However, while the investment cost flow rate is fully recovered as the product cost flow rate, the loss cost flow rate is discharged into the environment as the waste cost flow rate.

As can be seen in Equations (19) and (20), the total cost-balance equation by the SPECO method for the combined cycle power plant becomes the Moran equation given in Equation (7) when applying the unit cost of loss for exergy destruction obtained by Equation (20). If a unit lost cost of −$1.10/GJ obtained from Equation (20) is applied to the SPECO method, the unit costs of electricity produced by gas turbines and steam turbines are $26.7/GJ and $37.8/GJ, respectively, so the calculated weighted average unit cost is approximately $29.3/GJ, as shown in Table 7b. SPECO’s electricity unit cost matches the electricity unit cost of $29.5/GJ calculated according to the Moran method. The MOPSA method calculates unit electricity costs similar to the Moran method, and the calculated unit loss cost is −$0.24/GJ. In this specific case, the unit electricity cost of the gas turbine according to the SPECO method was slightly lower than that of the MOPSA method, and the unit electricity cost of the steam turbine according to the SPECO method was slightly higher than that of the MOPSA method.

Table 8a shows the cost flow rates for various exergies, loss cost flow, and investment cost flow rates for the gas turbine power plant using the MOPSA method. The flow rate of lost costs incurred in gas turbine power plants is much greater than the flow rate of lost costs incurred in combined cycle power plants. In this case, it can be seen that the lost cost flow of $4.35/s is caused by thermal and mechanical exergy leaking from the gas turbine power plant into the environment.

For a gas turbine power plant, the electricity unit cost evaluated using the SPECO method without considering lost cost is approximately $25.7/GJ, as can be seen in Table 8b. The total exergy destruction for the gas turbine plant is approximately 374.6 MW. On the other hand, the electricity unit costs evaluated by MOPSA and Moran are $39.0 and $38.5/GJ, respectively, which are much higher than the unit cost evaluated by SPECO without lost cost. The unit loss cost due to exergy destruction was calculated to be approximately −$11.6/GJ, which corresponds to a loss cost flow rate of −$4.35/s in the MOPSA method. This loss cost flow rate corresponds to approximately 45% of the investment, maintenance, and fuel input cost flow rate of −$9.64/s. For the SPECO method, the following auxiliary equation can be used to obtain the unit lost cost for the gas turbine plant.

(26) $$c_{T}{\dot{E}}_{x,606}^{T}+c_{P}{\dot{E}}_{x,606}^{P}+c_{S}\sum_{k}{\dot{E}}_{x<k>}^{D}=0$$

This equation indicates that all waste cost streams leaving the dissipation unit must be allocated to each component of the plant as a loss cost flow rate, as studied by Lugo-Méndez et al. [28]. Reference material regarding the calculation of waste cost flow rates in dissipation units and the method of allocating them to system components is presented by Uysal and Kwak [18]. The unit costs of thermal and mechanical exergy at state point 606 are approximately $34.2/GJ and $28.7/GJ, respectively. The total exergy destruction rate of the gas turbine plant is 374.6 MW. The SPECO method with Equation (26) provides a unit electricity cost of $38.4/GJ and unit lost cost of −$12.8/GJ, as shown in Table 8b. In addition, as can be seen in Table 7b and Table 8b, it was found that as the loss cost flow increases, it has an increasingly significant impact on the unit cost of electricity.

Table 9a shows the cost flow rates for various exergies, loss cost flow, and investment cost flow rates for a gas turbine cogeneration plant consisting of an air compressor, a combustor, a gas turbine, and a heat recovery steam generator by MOPSA. A pump unit was added to the cogeneration power plant, and the flow rate of water entering the HRSG was assumed to be 54.4 kmol/s. The total exergy destruction rate for the cogeneration plant is approximately 394.1 MW. In this particular case, the loss cost flow rate of $4.83/s due to exergy destruction is much higher than that of combined cycle power plants, but it is almost recovered by the steam production cost flow rate of $4.45/s. This loss cost flow is recovered through steam production in the HRSG, increasing the plant’s efficiency.

As can be seen in Table 7b, Table 8b and Table 9b, the difference in loss cost flow rates using the modified SPECO method and MOPSA method for combined cycle power plants, gas turbine power plants, and cogeneration power plants is 0.38, 0.04, and 0.66 $/s, respectively, which is not a significant difference. Furthermore, when loss costs are included, the electricity unit costs calculated by the Moran, SPECO, and MOPSA methods are very similar to one another.

For the SPECO method, the unit lost cost can be obtained from the following equation for the cogeneration plant.

(27) $$c_{607}{\dot{E}}_{x,607}+c_{503}{\dot{E}}_{x,503}+c_{S}\sum_{k}{\dot{E}}_{x<k>}^{D}=0$$

The unit cost of flue gas at the state point (607) is $38.0/GJ and the unit cost of water stream at the exit of HRSG (503) is $48.7/GJ. The total exergy destruction rate for the cogeneration plant was taken as 386 MW. The unit cost of electricity and steam obtained through SPECO is approximately $25.5/GJ and $30.8/GJ, respectively, without lost cost. However, as shown in Table 9b, the SPECO method with Equation (27) provides electricity and steam unit costs of $39.3/GJ and $50.1/GJ, respectively. The unit cost of electricity calculated by MOPSA is about $39.5/GJ, and the loss cost due to exergy destruction is −$12.1/GJ, which is similar to that of a gas turbine power plant. The unit cost of steam at the HRSG outlet calculated by MOPSA is approximately $43.9/GJ, and the obtained steam exergy flow is approximately 103.2 MW at a temperature of 435.0 K and a pressure of 1.95 MPa. The total loss cost flow of a gas turbine cogeneration plant reaches up to $4.84 per second, accounting for approximately 45% of the cost flow resulting from equipment, maintenance, and fuel costs.

Using the unit costs of various exergies obtained by MOPSA, which are shown in Table 6b, the unit cost at various state points of the combined cycle plant can also be calculated. The unit cost at state point 102 can be calculated as follows.

(28) $$c_{102}{\dot{E}}_{x,102}=c_{T}{\dot{E}}_{x,102}^{T}+c_{1P}{\dot{E}}_{x,102}^{P}$$

Table 10 presents the unit exergy costs at various state points calculated using the SPECO method (accounting for lost costs) and the values calculated using the MOPSA method for the combined cycle plant. It can be seen that the unit exergy costs calculated at various state points using the SPECO and MOPSA methods are very similar to each other except for the value at state point 552, which indicates that if the two exergoconomic methods appropriately distribute the unit cost of exergy destruction, they yield similar results not only for the unit exergy costs at all state points but also for the unit cost of exergy flow. The significantly lower unit cost at state points 552 and 562 based on MOPSA is due to the lower temperature of the water stream, which results in a lower exergy value at the state points 552 and 562. Meanwhile, as shown in Equations (14) and (15) of the SPECO method, the unit costs at state points 552 and 562 are determined by the unit costs at other state points.

## 6. Conclusions

This study systematically compared the unit cost of electricity obtained from different exergoeconomic methods—namely the Moran, SPECO, and MOPSA methods—across various power plant configurations, including gas turbine, cogeneration, and combined cycle systems. For a gas turbine power plant producing a single output—electricity—the unit costs of electricity calculated using the Moran, SPECO, and MOPSA methods were found to be $38.5/GJ, $25.7/GJ, and $39.0/GJ, respectively.

In this study, the SPECO method was modified by allocating the waste streams discharged from the dissipation units to each component as a lost cost associated with exergy destruction. This modification makes the overall cost-balance equation consistent with that of Moran’s equation. In contrast, the MOPSA method inherently accounts for exergy destruction during its formulation. Furthermore, since the total cost-balance equation becomes identical to Moran’s equation, MOPSA method yields the same unit costs as those calculated using Moran’s method.

For gas turbine power plants, the unit costs of electricity calculated using the Moran, modified SPECO, and MOPSA methods were $38.5/GJ, $38.4/GJ, and $39.0/GJ, respectively, showing remarkably close agreement. The loss cost flow rates calculated using the modified SPECO and MOPSA methods are −$4.79/s and −$4.83/s, respectively, corresponding to a difference of about $0.04/s. For a 300 MW combined cycle power plant, the weighted average unit costs of electricity using the Moran, modified SPECO, and the MOPSA methods were 29.5, 29.4, and 29.6 $/GJ, respectively. These results also show excellent agreement among the three methods. For this plant, the loss cost flow rates calculated using the modified SPECO and MOPSA methods were −$0.487/s and −$0.104/s, respectively, resulting in a difference of approximately $0.383/s.

These calculation results clearly demonstrate that the accurate allocation of lost cost associated with exergy destruction is essential for obtaining meaningful and consistent unit cost evaluations and lost cost flow rate. This can be accomplished by ensuring that the overall cost-balance equation for the system in question—regardless of the specific exergoeconomic method used—takes the form of the Moran equation.

In the modified SPECO and MOPSA methods, the overall cost-balance equation becomes identical to Moran’s equation, but the allocation of waste streams differs significantly between the two methods. In the MOPSA method, the “boundary” component serves discharge the waste cost flows associated with exergy destruction from the power plant to the environment. In the modified SPECO method, meanwhile, the waste flow leaving the dissipation unit represents the lost cost flow rate to the environment. Attempts were made to calculate identical production costs using different exergoeconomic methodologies; however, discrepancies emerged in the unit production cost of steam for the cogeneration plant.

Further research is required for cases involving cogeneration plants that generate both electricity and steam, or for scenarios involving changes in operating conditions.

## Figures and Tables

**Table 1:** Property values, calculated enthalpy and entropy flow rates, and thermal and mechanical exergy flows at various state points in the combined cycle plant at 100% load condition.

States	$\dot{n}$ [kmol/s]	T [K]	P [kPa]	$\dot{H}\left[\frac{\mathrm{kJ}}{s}\right]$	$\dot{S}$[kJ/sK]	${\dot{E}}_{x}^{T}$ [kJ/s]	${\dot{E}}_{x}^{P}$ [kJ/s]
101	15.0	288.2	103.3	6546.7	102.1	25.3	718.1
102	15.0	684.1	1609.4	185,426.2	148.2	63,015.5	102,872.2
303	0.5282	294.3	2812.8	420.6	−12.1	0.5	4351.9
304	0.5282	383.2	2812.8	2365.3	−6.3	228.9	4351.9
501/512	2.171	810.9	10,326.7	135,559.4	262.1	57,196.4	400.1
502	2.171	562.2	1757.7	117,591.8	265.7	38,484.2	64.9
503/508	0.293	424.1	4921.0	3371.5	9.7	465.3	25.5
504/505	0.293	328.2	4834.0	1236.4	4.0	31.1	25.0
506/571	3.041	388.7	175.8	26,569.0	81.1	2645.5	4.1
511	3.041	565.2	1757.7	165,079.0	372.8	54,079.3	91.0
514/521	3.041	791.7	1687.4	192,342.2	414.4	68,937.4	87.1
515/574	0.870	389.5	1971.5	7665.5	23.3	767.0	29.4
516	0.870	546.1	1757.7	46,502.2	105.3	15,138.7	26.0
517/582	2.171	390.8	10,897.6	19,617.3	58.4	1959.6	422.5
518	0.430	438.4	168.7	21681.1	57.5	4564.4	0.523
519/562	3.474	306.2	316.4	8679.1	29.9	27.9	13.5
522	3.041	504.4	175.8	169,745.5	421.6	35,280.0	4.1
541	3.470	493.3	168.7	182,070.0	479.6	39,356.4	4.2
542/543	3.470	306.4	5.1	150,012.5	491.2	3843.4	−6.0
544/561	3.470	306.2	5.1	8642.3	29.9	27.6	−6.0
551	279.2	283.2	211.0	212,242.4	759.3	8221.3	553.2
552	279.2	290.0	210.0	356,505.1	1262.8	2386.5	548.1
572	3.041	388.7	175.8	26,569.0	81.1	2645.5	4.1
581	2.171	389.5	1971.5	19141.8	58.1	1915.3	83.3
605	15.535	1509.2	1609.9	763,333.9	665.0	463,790.8	106,504.6
606	15.535	830.1	107.1	310,948.4	620.3	129,080.5	2134.6
607	15.535	410.0	105.1	72,084.9	224.6	8947.9	1408.7

**Table 2:** Exergy-balance equations for each component of the combined cycle plant. The exergy destruction rate by the Gouy–Stodola theorem for each component is shown in the third column.

Component	Exergy-Balance Equation	Exergy Destruction $E_{x,<k>}^{D}$
AC	${\dot{E}}_{x,101}-{\dot{E}}_{x,102}={\dot{E}}_{x<\mathrm{AC}>}^{W}+{\dot{E}}_{x,<\mathrm{AC}>}^{D}$	$T_{0}\left[{\dot{S}}_{102}-{\dot{S}}_{101}-{\dot{Q}}_{<\mathrm{AC}>}/T_{0}\right]$
COM	${\dot{E}}_{x,102}+{\dot{E}}_{x,304}+\mathrm{LH}V_{<\mathrm{COM}>}-{\dot{E}}_{x,605}={\dot{E}}_{x,<\mathrm{COM}>}^{D}$	$T_{0}\left[{\dot{S}}_{605}-{\dot{S}}_{102}-{\dot{S}}_{304}-{\dot{Q}}_{<\mathrm{COM}>}/T_{o}\right]$
GT	${\dot{E}}_{x,605}-{\dot{E}}_{x,606}={\dot{E}}_{x,<\mathrm{GT}>}^{W}+{\dot{E}}_{x,<\mathrm{GT}>}^{D}$	$T_{0}\left[{\dot{S}}_{606}-{\dot{S}}_{605}-{\dot{Q}}_{<\mathrm{GT}>}/T_{o}\right]$
FPH	${\dot{E}}_{x,303}-{\dot{E}}_{x,304}+{\dot{E}}_{x,503}-{\dot{E}}_{x,504}={\dot{E}}_{x,<\mathrm{FPH}>}^{D}$	$T_{0}\left[{\dot{S}}_{304}-{\dot{S}}_{303}+{\dot{S}}_{503}-{\dot{S}}_{504}-{\dot{Q}}_{<\mathrm{FPH}>}/T_{o}\right]$
ST	${\dot{E}}_{x,501}-{\dot{E}}_{x,502}+{\dot{E}}_{x,521}-{\dot{E}}_{x,522}+{\dot{E}}_{x,541}-{\dot{E}}_{x,542}$ $={\dot{E}}_{x,<\mathrm{ST}>}^{W}+{\dot{E}}_{x,<\mathrm{ST}>}^{D}\;$	$T_{0}\left[\begin{array}{c} {\dot{S}}_{502}-{\dot{S}}_{501}+{\dot{S}}_{522}-{\dot{S}}_{521}\\ +{\dot{S}}_{542}-{\dot{S}}_{541}-{\dot{Q}}_{<\mathrm{ST}>}/T_{o} \end{array}\right]$
CON	${\dot{E}}_{x,543}-{\dot{E}}_{x,544}+{\dot{E}}_{x,551}-{\dot{E}}_{x,552}={\dot{E}}_{x,<\mathrm{CON}>}^{D}$	$T_{0}\left[{\dot{S}}_{544}-{\dot{S}}_{543}+{\dot{S}}_{552}-{\dot{S}}_{551}-{\dot{Q}}_{<\mathrm{CON}>}/T_{o}\right]$
CP	${\dot{E}}_{x,561}-{\dot{E}}_{x,562}={\dot{E}}_{x,<\mathrm{CP}>}^{W}+{\dot{E}}_{x,<\mathrm{CP}>}^{D}$	$T_{0}\left[{\dot{S}}_{562}-{\dot{S}}_{561}-{\dot{Q}}_{<\mathrm{CP}>}/T_{o}\right]$
FPA	${\dot{E}}_{x,571}-{\dot{E}}_{x,572}={\dot{E}}_{x,<\mathrm{FPA}>}^{W}+{\dot{E}}_{x,<\mathrm{FPA}>}^{D}$	$T_{0}\left[{\dot{S}}_{572}-{\dot{S}}_{571}-{\dot{Q}}_{<\mathrm{FPA}>}/T_{o}\right]$
FPB	${\dot{E}}_{x,581}-{\dot{E}}_{x,582}={\dot{E}}_{x,<\mathrm{FPB}>}^{W}+{\dot{E}}_{x,<\mathrm{FPB}>}^{D}$	$T_{0}\left[{\dot{S}}_{582}-{\dot{S}}_{581}-{\dot{Q}}_{<\mathrm{FPB}>}/T_{o}\right]$
HRSG	${\dot{E}}_{x,606}-{\dot{E}}_{x,607}+{\dot{E}}_{x,505}+{\dot{E}}_{x,511}+{\dot{E}}_{x,515}+{\dot{E}}_{x,517}+{\dot{E}}_{x,519}\;$ $-{\dot{E}}_{x,506}-{\dot{E}}_{x,508}-{\dot{E}}_{x,512}-{\dot{E}}_{x,514}-{\dot{E}}_{x,516}-{\dot{E}}_{x,518}={\dot{E}}_{x,<\mathrm{HRSG}>}^{D}$	$T_{0}\left[\begin{array}{c} {\dot{S}}_{607}-{\dot{S}}_{606}+{\dot{S}}_{506}+{\dot{S}}_{508}+{\dot{S}}_{512}+{\dot{S}}_{514}\\ +{\dot{S}}_{516}+{\dot{S}}_{518}-{\dot{S}}_{505}-{\dot{S}}_{511}-{\dot{S}}_{515}+{\dot{S}}_{517}\\ +{\dot{S}}_{519}-{\dot{Q}}_{<\mathrm{HRSG}>}/T_{o} \end{array}\right]$

**Table 3:** Cost-balance equations for each component of the combined cycle plant by SPECO method.

Component	Cost-Balance Equation	Auxiliary Equation
AC	$c_{101}{\dot{E}}_{x,101}-c_{102}{\dot{E}}_{x,102}-c_{<\mathrm{AC}>}E_{x,<\mathrm{AC}>}^{W}=c_{S}E_{x,<\mathrm{AC}>}^{D}-{\dot{Z}}_{<\mathrm{AC}>}$	*c*_101_ = 0
COM	$c_{102}{\dot{E}}_{x,102}+c_{304}{\dot{E}}_{x,304}-c_{605}{\dot{E}}_{x,605}=c_{S}{\dot{E}}_{x,<\mathrm{COM}>}^{D}-{\dot{Z}}_{<\mathrm{COM}>}$	
GT	$c_{605}{\dot{E}}_{x,605}-c_{606}{\dot{E}}_{x,606}-c_{<\mathrm{GT}>}{\dot{E}}_{x,<\mathrm{GT}>}^{W}=c_{S}{\dot{E}}_{x,<\mathrm{GT}>}^{D}-{\dot{Z}}_{<\mathrm{GT}>}$	*c*_606_ = *c*_605_ (*fuel rule*) *c*_<*AC*>_ = *c*_<*GT*>_ (*product rule*)
FPH	$c_{303}{\dot{E}}_{x,303}-c_{304}{\dot{E}}_{x,304}+c_{503}{\dot{E}}_{x,503}-c_{504}{\dot{E}}_{x,504}=c_{S}{\dot{E}}_{x,<\mathrm{FPH}>}^{D}-{\dot{Z}}_{<\mathrm{FPH}>}$	*c*_504_ = *c*_503_ (*fuel rule*) $c_{303}=20.0\frac{\$}{\mathrm{GJ}}$
ST	$c_{501}{\dot{E}}_{x,501}-c_{502}{\dot{E}}_{x,502}+c_{521}{\dot{E}}_{x,521}-c_{522}{\dot{E}}_{x,522}+c_{541}{\dot{E}}_{x,541}-c_{542}{\dot{E}}_{x,542}$ $-c_{<\mathrm{ST}>}{\dot{E}}_{x,<\mathrm{ST}>}^{W}=c_{S}{\dot{E}}_{x,<\mathrm{ST}>}^{D}-{\dot{Z}}_{<\mathrm{ST}>}\;$	*c*_502_ = *c*_501_ (*fuel rule*) *c*_522_ = *c*_521_ (*fuel rule*) *c*_542_ = *c*_541_ (*fuel rule*)
CON	$c_{543}{\dot{E}}_{x,543}-c_{544}{\dot{E}}_{x,544}+c_{551}{\dot{E}}_{x,551}-c_{552}{\dot{E}}_{x,552}=c_{S}{\dot{E}}_{x,<\mathrm{CON}>}^{D}-{\dot{Z}}_{<\mathrm{CON}>}$	*c*_544_ = *c*_543_ (*fuel rule*) *c*_551_ = 0
CP	$c_{561}{\dot{E}}_{x,561}-c_{562}{\dot{E}}_{x,562}-c_{<\mathrm{CP}>}{\dot{E}}_{x,<\mathrm{CP}>}^{W}=c_{S}{\dot{E}}_{x,<\mathrm{CP}>}^{D}-{\dot{Z}}_{<\mathrm{CP}>}$	*c*_<*CP*>_ = *c*_<*ST*>_ (*product rule*)
FPA	$c_{571}{\dot{E}}_{x,571}-c_{572}{\dot{E}}_{x,572}-c_{<\mathrm{FPA}>}{\dot{E}}_{x,<\mathrm{FPA}>}^{W}=c_{S}{\dot{E}}_{x,<\mathrm{FPA}>}^{D}-{\dot{Z}}_{<\mathrm{FPA}>}$	*c*_<*FPA*>_ = *c*_<*ST*>_ (*product rule*)
FPB	$c_{581}{\dot{E}}_{x,581}-c_{582}{\dot{E}}_{x,582}-c_{<\mathrm{FPB}>}{\dot{E}}_{x,<\mathrm{FPB}>}^{W}=c_{S}{\dot{E}}_{x,<\mathrm{FPB}>}^{D}-{\dot{Z}}_{<\mathrm{FPB}>}$	*c*_<*FPB*>_ = *c*_<*ST*>_ (*product rule*)
HRSG	$c_{606}{\dot{E}}_{x,606}-c_{607}{\dot{E}}_{x,607}+c_{505}{\dot{E}}_{x,505}+c_{511}{\dot{E}}_{x,511}+c_{515}{\dot{E}}_{x,515}\;$ $+c_{517}{\dot{E}}_{x,517}+c_{519}{\dot{E}}_{x,519}-c_{506}{\dot{E}}_{x,506}-c_{508}{\dot{E}}_{x,508}-c_{512}{\dot{E}}_{x,512}\;$ $-c_{514}{\dot{E}}_{x,514}-c_{516}{\dot{E}}_{x,516}-c_{518}{\dot{E}}_{x,518}=c_{S}{\dot{E}}_{x,<\mathrm{HRSG}>}^{D}-{\dot{Z}}_{<\mathrm{HRSG}>}$	*c*_607_ = *c*_606_ (*fuel rule*) *c*_506_ = *c*_508_ = *c*_512_ = *c*_514_ = *c*_516_ = *c*_518_ (*product rule*)

*c*_505_ = *c*_504_, *c*_506_ = *c*_571_,*c*_508_ = *c*_503_, *c*_512_ = *c*_501_, *c*_521_ = *c*_514_, *c*_541_ = *c*_522_, *c*_543_ = *c*_542_, *c*_561_ = *c*_544_, *c*_572_ = *c*_515_, *c*_582_ = *c*_517_, *c*_562_ = *c*_519_, *c*_511_ = *c*_502_ (*same flow*).

**Table 4:** Cost-balance equations for each component of the combined cycle plant by MOPSA method.

Component	Cost-Balance Equations
AC	$\left({\dot{E}}_{x,101}^{T}-{\dot{E}}_{x,102}^{T}\right)c_{T}+\left({\dot{E}}_{x,101}^{P}-{\dot{E}}_{x,102}^{P}\right)c_{1P}-{\dot{E}}_{x<\mathrm{AC}>}^{W}c_{W}={\dot{E}}_{x,<\mathrm{AC}>}^{D}c_{S}-{\dot{Z}}_{<\mathrm{AC}>}$
COM	$\left({\dot{E}}_{x,102}^{T}+{\dot{E}}_{x,304}^{T}+\mathrm{LH}V_{<\mathrm{COM}>}-{\dot{E}}_{x,605}^{T}\right)c_{2T}+\left({\dot{E}}_{x,102}^{P}+{\dot{E}}_{x,304}^{P}-{\dot{E}}_{x,605}^{P}\right)c_{P}=c_{S}{\dot{E}}_{x,<\mathrm{COM}>}^{D}-{\dot{Z}}_{<\mathrm{COM}>}$
GT	$\left({\dot{E}}_{x,605}^{T}-{\dot{E}}_{,606}^{T}\right)c_{T}+\left({\dot{E}}_{x,605}^{P}-{\dot{E}}_{,606}^{P}\right)c_{P}-{\dot{E}}_{x,<\mathrm{GT}>}^{W}c_{3W}={\dot{E}}_{x,<\mathrm{GT}>}^{D}c_{S}-{\dot{Z}}_{<\mathrm{GT}>}$
FPH	$\left({\dot{E}}_{x,303}^{T}-{\dot{E}}_{x,304}^{T}\right)c_{4T}+\left({\dot{E}}_{x,303}^{P}-{\dot{E}}_{x,304}^{P}\right)c_{P}+\left({\dot{E}}_{x,503}^{\mathrm{DT}}-{\dot{E}}_{x,504}^{\mathrm{DT}}\right)c_{\mathrm{DT}}+\left({\dot{E}}_{x,503}^{\mathrm{DP}}-{\dot{E}}_{x,504}^{\mathrm{DP}}\right)c_{\mathrm{DP}}={\dot{E}}_{x,<\mathrm{FPH}>}^{D}c_{S}-{\dot{Z}}_{<\mathrm{FPH}>}$
ST	$\left({\dot{E}}_{x,501}^{\mathrm{DT}}-{\dot{E}}_{x,502}^{\mathrm{DT}}+{\dot{E}}_{x,521}^{\mathrm{DT}}-{\dot{E}}_{x,522}^{\mathrm{DT}}+{\dot{E}}_{x,541}^{\mathrm{DT}}-{\dot{E}}_{x,542}^{\mathrm{DT}}\right)c_{\mathrm{DT}}+\;$ $\left({\dot{E}}_{x,501}^{\mathrm{DP}}-{\dot{E}}_{x,502}^{\mathrm{DP}}+{\dot{E}}_{x,521}^{\mathrm{DP}}-{\dot{E}}_{x,522}^{\mathrm{DP}}+{\dot{E}}_{x,541}^{\mathrm{DP}}-{\dot{E}}_{x,542}^{\mathrm{DP}}\right)c_{\mathrm{DP}}-{\dot{E}}_{x,<\mathrm{ST}>}^{W}c_{5\mathrm{DW}}={\dot{E}}_{x,<\mathrm{ST}>}^{D}c_{S}-{\dot{Z}}_{<\mathrm{ST}>}$
CON	$\left({\dot{E}}_{x,543}^{\mathrm{DT}}-{\dot{E}}_{x,544}^{\mathrm{DT}}\right)c_{\mathrm{DT}}+\left({\dot{E}}_{x,543}^{\mathrm{DP}}-{\dot{E}}_{x,544}^{\mathrm{DP}}\right)c_{\mathrm{DP}}+\left({\dot{E}}_{x,551}^{\mathrm{DT}}-{\dot{E}}_{x,552}^{\mathrm{DT}}\right)c_{6\mathrm{DT}}+\left({\dot{E}}_{x,551}^{\mathrm{DP}}-{\dot{E}}_{x,552}^{\mathrm{DP}}\right)c_{P}={\dot{E}}_{x,<\mathrm{CON}>}^{D}c_{S}-{\dot{Z}}_{<\mathrm{CON}>}$
CP	$\left({\dot{E}}_{x,561}^{\mathrm{DT}}-{\dot{E}}_{x,562}^{\mathrm{DT}}\right)c_{\mathrm{DT}}+\left({\dot{E}}_{x,561}^{\mathrm{DP}}-{\dot{E}}_{x,562}^{\mathrm{DP}}\right)c_{7\mathrm{DP}}-{\dot{E}}_{x,<\mathrm{CP}>}^{W}c_{\mathrm{DW}}={\dot{E}}_{x,<\mathrm{CP}>}^{D}c_{S}-{\dot{Z}}_{<\mathrm{CP}>}$
FPA	$\left({\dot{E}}_{x,571}^{\mathrm{DT}}-{\dot{E}}_{x,572}^{\mathrm{DT}}\right)c_{\mathrm{DT}}+\left({\dot{E}}_{x,571}^{\mathrm{DP}}-{\dot{E}}_{x,572}^{\mathrm{DP}}\right)c_{8\mathrm{DP}}-{\dot{E}}_{x,<\mathrm{FPA}>}^{W}c_{\mathrm{DW}}={\dot{E}}_{x,<\mathrm{FPA}>}^{D}c_{S}-{\dot{Z}}_{<\mathrm{FPA}>}$
FPB	$\left({\dot{E}}_{x,581}^{\mathrm{DT}}-{\dot{E}}_{x,582}^{\mathrm{DT}}\right)c_{\mathrm{DT}}+\left({\dot{E}}_{x,581}^{\mathrm{DP}}-{\dot{E}}_{x,582}^{\mathrm{DP}}\right)c_{9\mathrm{DP}}-{\dot{E}}_{x,<\mathrm{FPB}>}^{W}c_{\mathrm{DW}}={\dot{E}}_{x,<\mathrm{FPB}>}^{D}c_{S}-{\dot{Z}}_{<\mathrm{FPB}>}$
HRSG	$\left({\dot{E}}_{x,606}^{T}-{\dot{E}}_{x,607}^{T}\right)c_{T}+\left({\dot{E}}_{x,606}^{P}-{\dot{E}}_{x,607}^{P}\right)c_{P}+\left({\dot{E}}_{x,505}^{\mathrm{DT}}+{\dot{E}}_{x,511}^{\mathrm{DT}}+{\dot{E}}_{x,515}^{\mathrm{DT}}+{\dot{E}}_{x,517}^{\mathrm{DT}}+{\dot{E}}_{x,519}^{\mathrm{DT}}\right)c_{\mathrm{DT}}\;$ $-\left({\dot{E}}_{x,506}^{\mathrm{DT}}+{\dot{E}}_{x,508}^{\mathrm{DT}}+{\dot{E}}_{x,512}^{\mathrm{DT}}+{\dot{E}}_{x,514}^{\mathrm{DT}}+{\dot{E}}_{x,516}^{\mathrm{DT}}+{\dot{E}}_{x,518}^{\mathrm{DT}}\right)c_{10\mathrm{DT}}+\left({\dot{E}}_{x,505}^{\mathrm{DP}}+{\dot{E}}_{x,511}^{\mathrm{DP}}+{\dot{E}}_{x,515}^{\mathrm{DP}}+{\dot{E}}_{x,517}^{\mathrm{DP}}+{\dot{E}}_{x,519}^{\mathrm{DP}}\right)c_{\mathrm{DP}}$ $-\left({\dot{E}}_{x,506}^{\mathrm{DP}}+{\dot{E}}_{x,508}^{\mathrm{DP}}+{\dot{E}}_{x,512}^{\mathrm{DP}}+{\dot{E}}_{x,514}^{\mathrm{DP}}+{\dot{E}}_{x,516}^{\mathrm{DP}}+{\dot{E}}_{x,518}^{\mathrm{DP}}\right)c_{\mathrm{DP}}={\dot{E}}_{x,<\mathrm{HRSG}>}^{D}c_{S}-{\dot{Z}}_{<\mathrm{HRSG}>}\;$
T-Junction	$\left({\dot{E}}_{x,102}^{T}+{\dot{E}}_{x,304}^{T}-{\dot{E}}_{x,605}^{T}+{\dot{E}}_{x,303}^{T}-{\dot{E}}_{x,304}^{T}\right)c_{T}=\left({\dot{E}}_{x,102}^{T}+{\dot{E}}_{x,304}^{T}-{\dot{E}}_{x,605}^{T}\right)c_{2T}+\left({\dot{E}}_{x,303}^{T}-{\dot{E}}_{x,304}^{T}\right)c_{3T}$
P-Junction	$\left({\dot{E}}_{x,101}^{P}-{\dot{E}}_{x,102}^{P}\right)c_{P}=\left({\dot{E}}_{x,101}^{P}-{\dot{E}}_{x,102}^{P}\right)c_{1P}$
DT-Junction	$\left({\dot{E}}_{x,551}^{\mathrm{DT}}-{\dot{E}}_{x,552}^{\mathrm{DT}}-{\dot{E}}_{x,506}^{\mathrm{DT}}+{\dot{E}}_{x,508}^{\mathrm{DT}}+{\dot{E}}_{x,512}^{\mathrm{DT}}+{\dot{E}}_{x,514}^{\mathrm{DT}}+{\dot{E}}_{x,516}^{\mathrm{DT}}+{\dot{E}}_{x,518}^{\mathrm{DT}}\right)c_{\mathrm{DT}}=\;$ $\left({\dot{E}}_{x,551}^{\mathrm{DT}}-{\dot{E}}_{x,552}^{\mathrm{DT}}\right)c_{6\mathrm{DT}}-\left({\dot{E}}_{x,506}^{\mathrm{DT}}+{\dot{E}}_{x,508}^{\mathrm{DT}}+{\dot{E}}_{x,512}^{\mathrm{DT}}+{\dot{E}}_{x,514}^{\mathrm{DT}}+{\dot{E}}_{x,516}^{\mathrm{DT}}+{\dot{E}}_{x,518}^{\mathrm{DT}}\right)c_{10\mathrm{DT}}$
DP-Junction	$\left({\dot{E}}_{x,561}^{\mathrm{DP}}-{\dot{E}}_{x,562}^{\mathrm{DP}}+{\dot{E}}_{x,571}^{\mathrm{DP}}-{\dot{E}}_{x,572}^{\mathrm{DP}}+{\dot{E}}_{x,581}^{\mathrm{DP}}-{\dot{E}}_{x,582}^{\mathrm{DP}}\right)c_{\mathrm{DP}}=\;$ $\left({\dot{E}}_{x,561}^{\mathrm{DP}}-{\dot{E}}_{x,562}^{\mathrm{DP}}\right)c_{7\mathrm{DP}}+\left({\dot{E}}_{x,571}^{\mathrm{DP}}-{\dot{E}}_{x,572}^{\mathrm{DP}}\right)c_{8\mathrm{DP}}+\left({\dot{E}}_{x,581}^{\mathrm{DP}}-{\dot{E}}_{x,582}^{\mathrm{DP}}\right)c_{9\mathrm{DP}}$
W-Junction	${\dot{E}}_{x,<\mathrm{GT}>}^{W}c_{W}={\dot{E}}_{x,<\mathrm{GT}>}^{W}c_{3W}$
DW-Junction	${\dot{E}}_{x,<\mathrm{ST}>}^{W}c_{\mathrm{DW}}=E_{x,<\mathrm{ST}>}^{W}c_{5\mathrm{DW}}$
Boundary	$\left({\dot{E}}_{x,101}^{T}+{\dot{E}}_{x,303}^{T}-{\dot{E}}_{,607}^{T}\right)c_{T}+\left({\dot{E}}_{x,101}^{P}+{\dot{E}}_{x,303}^{P}-{\dot{E}}_{,607}^{P}\right)c_{P}\;$ $\left({\dot{E}}_{x,551}^{\mathrm{DT}}-{\dot{E}}_{,552}^{\mathrm{DT}}\right)c_{\mathrm{DT}}+\left({\dot{E}}_{x,551}^{\mathrm{DP}}-{\dot{E}}_{x,552}^{\mathrm{DP}}\right)c_{\mathrm{DP}}+T_{o}\left({\dot{S}}_{101}+{\dot{S}}_{303}-{\dot{S}}_{607}+{\dot{S}}_{551}-{\dot{S}}_{552}+\sum_{k}{\dot{Q}}_{<k>}/T_{o}\right)c_{s}=0$

**Table 5:** Calculated exergy streams for each component of the combined cycle plant.

Component	${\dot{E}}_{x}^{W}$ (kW)	${\dot{E}}_{x}^{T}$ (kW)	${\dot{E}}_{x}^{P}$ (kW)	Irreversibility Rate (kW)	
				$T_{o}\left({\dot{S}}_{\mathrm{out}}-{\dot{S}}_{\mathrm{in}}\right)$	$-{\dot{Q}}_{<k>}$
AC	−178,879.5	62,990.3	102,154.0	13,735.3	0
COM	−731,205.5 *	400,546.4	−719.4	155,989.5	175,389.0
GT	429,344.8	−334,710.4	−104,370.0	−13,305.2	23,040.8
FPH		−205.9	−0.5	15.9	190.4
ST	81,621.8	−87,882.6	−428.5	6689.2	0
COND		−9650.6	−5.0	12,548.2	−2892.6
PC	−28.0	0.3	19.5	8.2	0
PFA	−248.8	39.2	98.6	111.0	0
PFB	−475.5	44.3	349.2	82.0	0
HRSG		−32,614.1	−1564.8	19,063.7	15,115.2
Total	331,334.8 (−731,205.5 *)	−1443.1	−4466.9	194,937.8	210,842.8

* Heat of reaction is modified for the exergy-balance.

**Table 7:** (**a**) Cost flow rates of various exergies, lost costs, and investment cost flow rates of each component of the combined cycle plant by MOPSA method. Unit is $/s. (**b**) Unit cost of electricity and lost work ($/GJ), and lost cost flow rate from various exergoeconomic methods for the combined power plant.

**(a)**					
**Component**	**Thermal**	**Mechan**	**Work**	**Lost Cost**	**Investment**
AC	1.51136	3.39592	−4.82172	−0.00336	−0.08220
GT	−8.03090	−3.46959	11.57304	−0.00238	−0.07017
COM	0.11943	−0.02392		−0.08589	−0.00962
FPH	9.48200	−0.00003		−0.00005	−9.48192
ST	−2.95395	−0.02787	3.07568	−0.00164	−0.09223
CON	0.01838	−0.00033		−0.00236	−0.01564
CP	0.00001	0.00217	−0.00106		−0.00112
FPA	0.00132	0.00961	−0.00938	−0.00003	−0.00152
FPB	0.00149	0.01861	−0.01792	−0.00002	−0.00217
HRSG	0.12114	−0.07869		−0.00836	−0.03408
Total	0.27028	−0.17412	9.79864	−0.10409	−9.79067
**(b)**					
**Method**	**Unit Cost of Electricity from Gas Turbine**	**Unit Cost of Electricity From Steam Turbine**	**Weighted** **Average Unit Cost of Electricity**	**Unit Cost of Lost Work/** **Lost Cost Flow Rate** **($/s)**	
Moran			29.5		
SPECO without lost cost	25.6	35.6	28.0		
SPECO with lost cost	26.7	37.8	29.4	−1.10/−0.487	
MOPSA	27.0	37.7	29.6	−0.24/−0.104	

**Table 8:** (**a**) Cost flow rates of various exergies, lost costs, and investment cost flow rates of each component of the gas turbine power plant by MOPSA method. Unit is $/s. (**b**) Unit cost of electricity and work ($/GJ) and lost cost flow rate and input cost flow rate from various exergoeconomic methods for the gas turbine power plant.

**(a)**					
**Component**	**Thermal**	**Mechan**	**Work**	**Lost Cost**	**Investment**
AC	2.1383	5.0729	−6.9695	−0.1594	−0.0822
COM	13.5969	−0.0357		−4.0757	−9.4855
GT	−11.3621	−5.1830	16.7282	−0.1130	−0.0702
Total	4.3731	−0.1458	9.7587	−4.3481	−9.6379
**(b)**					
**Method**	**Unit Cost of Electricity from Gas Turbine**		**Unit Cost of Lost Work**	**Lost Cost Flow Rate/Input Cost Flow Rate ($/s)**	
Moran	38.5				
SPECO without lost cost	25.7				
SPECO with lost cost	38.4		−12.8	−4.79/−9.64	
MOPSA	39.0		−11.6	−4.83/−9.64	

**Table 9:** (**a**) Cost flow rates of various exergies, lost costs, and investment cost flow rates of each component of the cogeneration plant by MOPSA method. Unit is $/s. (**b**) Unit cost of electricity ($/GJ), steam and lost work, and lost cost flow rate and input cost flow rate from various exergoeconomic methods for the cogeneration plant.

**(a)**						
**Component**	**Thermal**	**Mechan**	**Work**	**Heat**	**Lost Cost**	**Investment**
AC	2.1683	5.1531	−7.0723		−0.1669	−0.0822
COM	13.7876	−0.0363			−4.2658	−9.4855
GT	−11.5214	−5.2649	16.9747		−0.1183	−0.0702
CP	0.0003	0.2784	−0.2297		−0.0479	−0.0011
HRSG	−4.1352	−0.0440		4.4499	−0.2366	−0.0341
Total	0.2996	0.0863	9.6727	4.4499	−4.8355	−9.6731
**(b)**						
**Method**	**Unit Cost of Electricity from Gas Turbine**		**Unit Cost of Steam**	**Unit Cost of Lost Work**	**Lost Cost Flow Rate/Input Cost Flow Rate** **($/s)**	
Moran	39.5					
SPECO without lost cost	25.5		30.8			
SPECO with lost cost	39.3		50.1	−13.8	−5.50/−9.67	
MOPSA	39.5		43.9	−12.1	−4.84/−9.67	

**Table 10:** Unit costs of material flow at various state points in the combined cycle power plant by SPECO/MOPSA methods (Unit is $/GJ) when the lost cost due to exergy destruction is taken into account. The numbers in parentheses indicate the percentage difference between the SPECO and MOPSA values.

***c*_1_(*c*_102_)**	***c*_2_(*c*_605_)**	***c*_3_(*c_W_*)**	***c*_4_(*c*_304_)**	***c*_5_(*c_DW_*)**
29.33/29.73(−1.36)	25.88/30.46(−17.7)	26.65/26.96(−1.16)	2073.1/2104.3(−1.50)	37.82/37.68(0.37)
***c*_6_(*c*_552_)**	***c*_7_(*c*_562_)**	***c*_8_(*c*_572_)**	***c*_9_(*c*_582_)**	***c*_10_(*c*_512_)**
32.31/12.30(61.9)	70.48/59.2(16.0)	35.86/36.17(−0.86)	36.51/37.10(−1.62)	33.55/34.27(−2.74)

## Data Availability

Data are not available for ethical reasons.

## Nomenclature

*c*	unit cost of exergy or material stream
*C_ini_*	initial investment cost
*c_o_*	unit cost of fuel
*C*	cost of equipment
$\dot{C}$	cost flow rate
*e_x_*	exergy per unit mass
${\dot{E}}_{x}$	exergy flow rate
${\dot{E}}_{x}^{D}$	exergy destruction rate
$\dot{H}$	enthalpy flow rate
$\dot{m}$	mass flow rate
*Q*	capacity of equipment
$\dot{Q}$	heat flow rate
*s*	entropy per unit mass
$\dot{S}$	entropy flow rate
${\dot{S}}_{\mathrm{gen}}$	entropy generation rate
*T*	temperature
*T_o_*	ambient temperature
$\dot{W}$	work flow rate
*X*	scaling exponent
$\dot{Z}$	capital cost flow rate
*Greek letters*	
*δ*	annual operating hours
*φ*	maintenance factor
*Subscripts*	
cv	control volume
E	electricity
o	ambient or reference
P	mechanical
ref	reference
S	entropy, entropy generation
T	thermal
W	work or electricity
x	exergy
*Superscripts*	
CHE	chemical
D	exergy destruction
P	mechanical exergy
T	thermal exergy
W	work or electricity
*Abbreviations*	
AC	air compressor
COM	combustor
CON	condenser
CRF	capital recovery factor
CP	condensate pump
EC	economizer
EV	evaporator
FP	feed pump
GSC	gland steam condenser
GT	gas turbine
GTG	gas turbine generator
HP	high-pressure
HRSG	heat recovery steam generator
IP	intermediate-pressure
LHV	lower heating value
LP	low-pressure
MOPSA	modified productive structure analysis
PPH	fuel preheater
PWF	present worth factor
RH	reheater
SH	superheater
SPECO	specific exergy costing
ST	steam turbine
STG	steam turbine generator
SV	salvage value

## References

[1] Howell J.R. (1987). Fundamentals of Engineering Thermodynamics.

[2] Bejan A. (1988). Advanced Engineering Thermodynamics.

[3] Oh S.D., Pang H.S., Kim S.M., Kwak H. (1996). Exergy analysis for a gas turbine cogeneration system. J. Eng. Gas. Turbines Power.

[4] Tribus M., Evans R. (1962). The Thermoeconomics of Seawater Conversion.

[5] Frangopoulos C.A. (1987). Thermo-economic functional analysis and optimization. Energy.

[6] von Spakovsky M.R., Evans R.B. (1993). Engineering functional analysis—Part I. J. Energy Resour. Technol..

[7] Valero A., Serra L., Lozano M.A., Richter H.J. (1993). Structural theory of thermoeconomics. Thermodynamics and the Design, Analysis and Improvement of Energy Systems.

[8] Lozano M.A., Valero A. (1993). Theory of the exergetic cost. Energy.

[9] Lazzaretto A., Tsatsaronis G. (1997). On the quest for objective equations in exergy costing. Proceedings of the ASME Advanced Energy Systems Division.

[10] Kwak H.Y., Kim D.J., Jeon J.S. (2003). Exergetic and thermoeconomic analysis of power plants. Energy.

[11] Lazzaretto A., Tsatsaronis G. (2006). SPECO: A systematic and general methodology for calculating efficiencies and cost in thermal systems. Energy.

[12] Orhan M.F., Dincer I., Rosen M.A. (2010). Exergoeconomic analysis of a thermochemical copper–chlorine cycle for hydrogen production using specific exergy cost (SPECO) method. Thermochim. Acta.

[13] Abusoglu A., Kanoglu M. (2009). Exergetic and thermoeconomic analysis of diesel engine powered cogeneration: Part 2—Application. Appl. Therm. Eng..

[14] Atmaca A., Yumrutas R. (2014). Thermodynamic and exergoeconomic analysis of a cement plant: Part II—Application. Energy Convers. Manag..

[15] He H., Liu X., Wang M., Zhou S., Heydarian D. (2022). Energy, exergy, exergoeconomic, and environmental analyses and multi-objective optimization of a biomass-to-energy integrated thermal power plant. Alex. Eng. J..

[16] Uysal C., Kurt H., Kwak H. (2017). Exergetic and thermoeconomic analyses of a coal-fired power plant. Int. J. Therm. Sci..

[17] Yilmaz C. (2018). Thermoeconomic cost analysis and comparison of methodologies for Dora II binary geothermal power plant. Geothermics.

[18] Uysal C., Kwak H. (2020). Role of waste cost in thermoeconomic analysis. Entropy.

[19] Hou S., Zhou Y., Yu L., Zhang F., Cao S., Wu Y. (2018). Optimization of a novel cogeneration system including a gas turbine, a supercritical CO2 recompression cycle, a steam power cycle and an organic Rankine cycle. Energy Convers. Manag..

[20] Oh H.S., Lee Y., Kwak H. (2017). Diagnosis of combined cycle power plant based on thermoeconomic analysis: A computational study. Entropy.

[21] Cengel Y.A., Boles M.A. (2011). Thermodynamics: An Engineering Approach.

[22] (1971). JANAF Thermochemical Tables.

[23] Japanese Society of Mechanical Engineers (1968). JSME Steam Tables.

[24] Kotas T.J. (1995). The Exergy Method of Thermal Plant Analysis.

[25] Kwak H., Byun G.T., Kwon Y.H., Yang H. (2004). Cost structure of CGAM cogeneration system. Int. J. Energy Res..

[26] Moran M.J. (1982). Availability Analysis: A Guide to Efficient Energy Use.

[27] Baumann C., Lopatnikov A. (2017). Scaling laws: Uses and misuses in industrial plant and equipment replacement cost estimates. MTS J..

[28] Lugo-Méndez H.D., Torres-González E.V., Castro-Hernández S., Salazar-Pereyra M., López-Arenas T., Lugo-Leyte R. (2020). An irreversibility-based criterion to determine the cost formation of residues in a three-pressure-level combined cycle. Entropy.

